# Credible pigeon permissioned blockchain traceability platform integrated with IoT based on HACCP

**DOI:** 10.1038/s41598-022-27065-2

**Published:** 2022-12-26

**Authors:** Mingyuan Fan, Shuangyin Liu, Longqin Xu, Dachun Feng, Jianjun Guo, Liang Cao, Tonglai Liu, Hassan Shahbaz Gul

**Affiliations:** 1 College of Information Science and Technology, Zhongkai University of Agriculture and Engineering, Guangzhou, 510225 China; 2 Smart Agriculture Engineering Technology Research Center of Guangdong Higher Education Institues, Zhongkai University of Agriculture and Engineering, Guangzhou, 510225 China; 3 Guangzhou Key Laboratory of Agricultural Product Quality, Safety Traceability Information Technology, Zhongkai University of Agriculture and Engineering, Guangzhou, 510225 China; 4 Guangdong Provincial Agricultural Products Safety Big Data Engineering Technology Research Center, Zhongkai University of Agriculture and Engineering, Guangzhou, 510225 China; 5 Guangdong Province Key Laboratory of Waterfowl Healthy Breeding, Guangzhou, 510225 China; 6 Academy of Smart Agricultural Engineering Innovations, Zhongkai University of Agriculture and Engineering, Guangzhou, 510225 China

**Keywords:** Engineering, Software

## Abstract

The pigeon food production industry from breeding to processing into food for market circulation involves many stages and people, which is prone to food safety issues and difficult to regulate. To address these problems, one possible solution is to establish a traceability system. However, in traditional traceability systems, a number of stages involved and each of them provides their own data accumulated in the database. Therefore, complex traceability data are compose of too many stages easily result in confusing information for customers. Besides, centralized data storage makes data vulnerable to be tampered with. To solve these problems, hazard analysis and critical control points (HACCP) principles have been utilized in our work which is a comprehensive traceability system. In this work, we analyze the pigeon food production industry through HACCP principles and determine some critical control points (CCPs), including incubation, breeding, transportation, slaughtering, processing, and logistics. With the help of these CCPs, we are able to build a traceability system with critical and abundant data but not too complicated for users. To further improve the system, there are different kinds of techniques integrated into it. Firstly, a permissioned blockchain, Hyperledger Fabric, is selected as blockchain module to enhance trustworthiness of data. Secondly, the system contains various IoT devices for automatically collecting environmental parameter data with the aim of reducing human interference. Besides, it is worth mentioning that the proposed information management device can decrease the data entry burden. Consequently, the implementation of the traceability system increase consumers’ confidence in pigeon food production. To summarize, it is a new application of modern agricultural information technique in food safety and a bold experiment in the field of poultry, particularly pigeons.

## Introduction

Livestock farming is a source of livelihood for billions of the world’s poor. The poultry sector is probably the most rapidly growing and flexible of all livestock sectors. Over the past 15 years, the demand for poultry has continued to grow in both developed and developing countries. According to the FAO survey, the growth in global meat protein consumption is expected to increase by an average of 14% over the next decade dominated by an increase consumption in poultry production, driven by the growth of income and population^1^. Chickens, ducks, geese, and pigeons are common types of poultry bred by people. Humans have a long history of raising pigeons for food, with the earliest written records of pigeons being raised for meat dating back to 60 AD in Spain^2^. Pigeon farming and the processed food industry have a huge market potential because pigeon meat is delicious and rich in nutrients such as protein, vitamins, calcium, and iron, with a 12–15% mortality rate, much lower than other poultry^3^. Pigeons are more simple to feed than most of other poultry and do not require particularly large premises for breeding. Moreover, they need a short breeding cycle and have a low incidence of disease. Therefore, it is an ideal choice to farm pigeons among poultry^4^. According to the current pigeon breeding technique, it requires a high labor cost.

Traditional pigeon farming is mostly free-range. This method of farming is inefficient in terms of labor and tends to increase the risk of poultry disease. The continuing aging of the world’s population has been an inevitable trend for a long time. According to the FAO report, between 1950 and 2015, children under five declined from 13.4 to 9.1%, while the proportion of older people over 65 will rise from 5.1% to 8.3%. By the end of this century, children under five could fall to 5.8%, while the proportion of older people is expected to rise to 22.7%^5^. So traditional pigeon farming is no longer suitable for rural realities in the context of an aging population. To increase the poultry breeding industry’s productivity due to a lack of labor force caused by aging in rural areas, MeiZhou, Guangdong Province in China has developed a large-scale, intensive meat pigeons breeding industry. Many pigeon lofts about 100 meters long and 20 meters wide with pigeon cages placed at designed distances have been built with the village as the unit and only one worker can raise and manage thousands of pigeons. A comfortable and relatively constant environment is essential for the healthy growth of the pigeons during their breeding. More precisely, abnormalities in the temperature^6^, humidity, noise, the length of light in the loft, harmful gases such as ammonia (usually fermented by pigeon feces), and other environmental parameters can interfere with pigeon’s egg-laying and hatching behavior, making them susceptible to epidemics and increasing the risk of pigeon breeding.

The traditional method to keep the pigeon breeding environment normal is manual measurement with regular use of instruments, but it is time-consuming and laborious especially in the case of more and more serious aging trend in rural areas. So sensors for monitoring environmental abnormalities have been installed in the pigeon loft to collect environmental data at regular intervals. By using sensors, the labor force requirement in pigeon breeding further decreases. Furthermore, the environmental data collected by sensors can be used for data analysis to predict the abnormal situation which may occur in the pigeon breeding in the future. Most of poultry will be used as raw materials of meat food for processing when they grow up. Besides, some slaughter and processing factories with a high degree of automation have been built in town not far from pigeon loft to implement the automation of slaughter, processing, and packaging of meat pigeon production. Finally, packaged meat pigeon food production will be transported to local hotels and supermarkets by trucks for customers to shop. All of these phases construct a pigeon food production industry from meat pigeon breeding to food production, which make efficient use of the agricultural labor force, and offering job opportunities for middle-aged and elder people. Among these phases, pigeon food production is a critical but weak phase because of frequently happened food safety scandal. Food safety is a major concern for consumers and have led to a crisis of confidence in the food-related industry especially in China^7^. But modern agricultural production and food processing do involve many people and complex processes. So it is necessary to find a mature ideology or theory to ensure food safety in pigeon food production industry and clarify responsibility lies.

Hazard analysis and critical control points(HACCP) is a management approach to food safety that was originally used to ensure the safety of food consumed by astronauts in space. Its basic idea is to analyse all the processes involved in the production and distribution of agricultural products to the consumer, to determine some critical control points(CCPs) that may pose food safety risks from a variety of perspectives, including biological and chemical. Based on these CCPs, supervisors can establish some critical limits to control unsafe operating conditions at a CCP. Then supervisors can use some monitoring procedures like traceability system to guarantee a CCP is under control and to gradually apply it to food safety for the general public^8^. From the pigeon loft to the consumer, meat pigeon products pass through several stages. Therefore, we use HACCP principles to analyze these stages and determine some CCPs such as egg production, breeding, transport, slaughter, processing and logistics as described previously and there are some critical data existing in each CCP. In addition, with the help of information technology, we can accurately persists data of each CCP in a database and establishes a traceability system. Through the traceability data provided by the system, consumers can clearly know the process of pigeon food production industry and enhance their confidence in food safety.

However, data stored in centralized databases have low credibility and can be easily tampered with without detecting by the outside world. On the other hand, the emergence of distributed storage technologies can greatly improve the trustworthiness and security of traceability data. Blockchain technology which originated in the Bitcoin white paper is a new type of distributed storage technology incorporating asymmetric encryption. Typically, blockchain exists in blockchain network and the network is compose of a number of distributed nodes. To guarantee data security, each node in blockchain network has its individual ledger with same copy of data. For this reason it is quite difficult to modify the data on ledger of each node at the same time. Another reason that data stored in the blockchain is hard to be tampered with is the Merkle tree^9^, which is a useful storage structure. Based on Bitcoin, various blockchains mainly divided into public blockchains and permissioned blockchains have emerged due to various needs of different business scenarios. One main differ between different types of blockchain is consensus mechanisms. For instance, public blockchains^10^ such as Bitcoin, Ethereum have relatively low transactions per second (TPS) because they use proof of work (PoW) consensus, which is belong to a power-hungry consensus mechanisms. On the other hand, permissioned blockchains usually use crashed fault tolerance (CFT) consensus mechanisms which need less computing power with the advantage of keeping relatively high TPS even facing large amount of website visiting. Hyperledger Fabric^11^ is a major representive of permissioned blockchain^11^ open-sourced by IBM which use Raft^12^ consensus mechanisms and possess a unique identity authentication mechanisms called membership service providers (MSP). MSP is a specialized identity authentication mechanisms defined on the basis of the x509 standard^13^. Its identity authentication materials are compose of certificate authority (CA) certificates, key pairs for asymmetric cryptographic communication and other identity proof materials to prevent the blockchain network from malicious nodes. Typically, traceability system must fulfill the basic requirement of storing large amount of data. Therefore, public blockchain may not be the best choice for traceability system. Apparently permissioned blockchain like Fabric is a better option.

To summarize, in order to prevent people from food safety issues and improve productivity of pigeon food production industry in an era of ageing population, we do some works from the perspective of agricultural information. More precisely, this research uses HACCP principles to analyze pigeon food production industry and then determine some CCPs. Based on these CCPs, we build a traceability system composed of different kinds of hardware device with various techniques. Keeping environment of pigeon breeding stable makes sense to reduce the risk of pigeon breeding. Therefore, we install some sensors to collect a variety of important environment data. Then we integrate environment data into our traceability system so that the monitoring of pigeon breeding environment becomes automatic, which reduces the workload of pigeon breeders. For further decreasing workforce required to deal with the tendency of ageing population, we design an auxiliary microcontroller device which can generate data by pressing buttons instead of recording data with keyboards. Assistance of the device not only reduces data entry workload of every breed but also avoids wrong data caused by breeder’s carelessness. Furthermore, we also integrate permissoned blockchain into the traceability system as data storage module to guarantee reliability of data.

The rest sections of this paper are organized as follows. “Literature review” section discusses the related works. “Permissioned blockchain and IoT based pigeon traceability system” section illustrates the proposed platform architecture and data flow. “System implementation and performance analysis” section details the implementation of the platform and system performance analysis. “Results and discussion” section discusses the limitations and indicates future research directions for the proposed system. ’Conclusion” section concludes the paper.

## Literature review

### Traditional centralize traceability system

Automation technology facilitates the development of agriculture and industrial technology increases the efficiency of food processing. But on the other hand, new technologies bring higher probability of occurrence of food safety issues. Therefore, it is necessary to select some technologies to handle food safety issues. Traceability technology is a typical and useful IT approach to prevent customers from food safety issues. Traceability is an ancient concept in food safety and the earliest traceability of food dated back 5000 years to Egypt^14^. Modern traceability refers to build a traceability system with information technology, which is beneficial for consumers to know information of both agricultural production and food processing processes clearly. Therefore, there are many researchers focus on how to build a traceability system with the purpose of increasing the production efficiency of the pigeon production industry. Web technique is a basic and widely used technique to construct a traceability system. Purnomo et al.^15^ established a poultry farming management and traceability system based on web technology with JSON as the data transfer format and MySQL as the database. By using the system, the poultry farming processes become more standardized and orderly, but the data stored in the centralized database is not highly credible and can be easily tampered with. What’s worse, complexity and tediousness of data entry may lead to some incorrect data inputted by breeders before they are stored by database. Therefore, how to decrease probability of wrong data entry in traceability system is a topic deserves to study. Zhou et al.^16^ used a handheld device to read the identity information of pigeons so that breeders can quickly access pigeon information while greatly reducing the possibility of recording incorrect data into the database. Besides, they created a website to visualize and manage pigeon information data, but they do not solve the problem of data being easily tampered with either. Stable and comfortable environment is quite important for poultry breeding because bad environment condition will lead to mass mortality of poultry. So more work is focus on keeping the breeding environment stable and monitoring the environment. Chai et al. installed sensors to monitor environment on pigeon lofts so that breeders can react environmental anomalies promptly through mobile phones or a large LED screen^17^. Esnaola-Gonzalez et al.^18^ also installed sensors to collect environment data on poultry farming premises with the aim of monitoring environment automatically. Furthermore, they divided poultry breeding industry into multiple processes such as breeding and slaughter. Based on these processes, they used a combination of relational and non-relational database to store both environment data and other critical data in every process. By recording data from multiple stages, the complex process of poultry production industry is no longer chaotic, but the centralized storage of relational and non-relational databases still allows the system owner to modify the data easily.

### Blockchain traceability system

Blockchain is a highly trusted distributed database which can greatly increase the trustworthiness from the perspective of data storage. However, because blockchain is a nascent technology, there are a few researches focus on integrating blockchain technology into agriculture especially food safety field. Investigations are significant for the public when a new technology may influence their daily life gradually like building a traceability system to ensure the safety of what they eat. Sander et al.^19^ gathered some data from several surveys and did some analyses according to these data. Based on results of these analyses, it can be concluded that consumers are not only willing to use blockchain-based meat transparency and traceability system (TTS) but also prefer to buy products with a traceability system using blockchain technology. Therefore, making use of blockchain technology more effective to protect people from food safety issues is worth researching. Some researchers have done some works about guaranteeing food safe by building a blockchain-based traceability system. Lin et al.^20^ proposed a food traceability system using blockchain incorporates IoT devices to help record data. However, they just proposed the architecture of the traceability system but they did not create a real website. Typically, features of website are defined by business logic and developed by programming languages. Similarly, features of blockchain-based website which exist in blockchain network are compose of various smart contracts developed by different kinds of blockchain’s individual API. Wang et al.^21^ used Fabric as a blockchain framework to build a food traceability system. More precisely, the system are compose of many smart contracts developed according to corresponding business logic. Smart contracts (also known as chaincode in Fabric) are not only use to control the nodes in the Fabric network but also can execute different business logic under specific conditions, i.e., in different parts of the food production industry such as production and processing. However, the lack of hardware support leads to cumbersome and error-prone data entry. Cao et al.^22^ used Ethereum to implement a traceability system combined with hardware devices for enhancing consumer’s confidence in food safety about Australian beef exported to China. However, obviously Ethereum is a kind of public blockchain so that it consumes much arithmetic power and has lower TPS than that of permissioned blockchain such as Fabric. Therefore, it is not the best choice to build a traceability system by using Ethereum from perspective of TPS because it is difficult for Ethereum to withstand concurrent writes with large data volumes. To summarize, the application of blockchain technology in agriculture is still in the exploratory stage. Among the existing work, few work is aim at developing a blockchain-based traceability system escpecially permissioned blockchain integrated with IoT. Furthermore, the application of agricultural information in the field of the pigeon food production industry is also rare. To fill in the gaps as described above, we propose and build a traceability system comprehensively uses permissioned blockchain and various hardware devices based on analyses of pigeon food production industry according to the HACCP principles.

## Permissioned blockchain and IoT based pigeon traceability system

### System architecture

**Figure 1:**
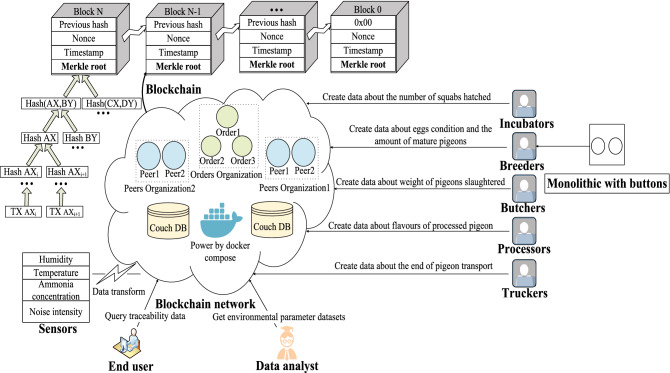
System architechture.

The architecture of the proposed traceability system is shown in Fig. 1. The system consists of several main components: a traceability data gathering module, a pigeon eggs information generation module, a sensor data collection module, a blockchain network, and a user terminal.

The kernel of the system is the blockchain network. There are some chain-connected blocks in the network for storing data which are ingeniously designed. As shown in the Fig. 1, the blocks contain randomly generated nonce numbers calculated by hash algorithm and time-based timestamp values. Both of them are not only a tool to verify the data do not be tampered with but also the difficulty of a puzzle that miners (nodes in blockchain network) need to find the correct generate value by computing power, aka mining. The hash value also plays a role in pointers for connecting blocks generated by Merkle tree. Merkle tree is a special storage structure which stores different data in every leaf node of the tree. It performs hash operations from the leaf node layer upwards the root node and every node in the tree gains a calculated value. The value of root node is the generated value we mentioned above, aka the pointer for connecting blocks. It can be concluded that once a block in the middle of blockchain is modified, the hash value as a pointer will inevitably change. Then the modification will cause all blocks from behind that block to fall out of the chain according to the irreversible one-way output character of the hash operations. In fact, if someone try to reconnect the chain, they have to correctly forge all random value afterward by calculation. However, the arithmetic power required to complete this process is too immense for one person or a couple of people to achieve. Therefore, it is nearly impossible for a malicious node in the blockchain network to record fake data into blockchain. It follows that the sophisticated structure of blockchain ensures high credibility of data in blockchain.

**Figure 2:**
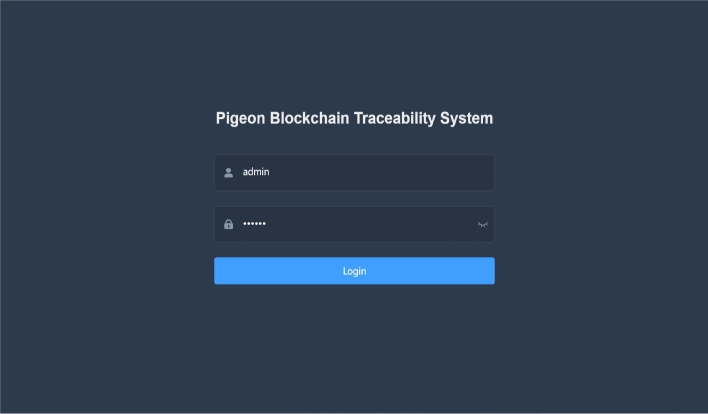
Login page.

**Figure 3:**
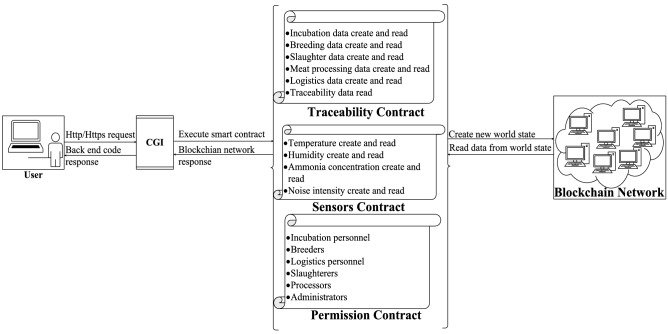
Smart contract architecture.

The Fabric blockchain network is different from that of other blockchain. In general, the network is driven by Docker. The topology of the network and role of the nodes are defined by the configuration files required by Docker-Compose. Nodes in the network can be divided into peer nodes and order nodes. A couple of nodes of same type can form a consortium, which is called an order consortium or a peering consortium according to corresponding type. Order nodes are responsible for ordering all transactions among the blockchain by timestamp. Then they store transactions into leaf nodes of Merkle tree and calculate root node value as describe above to construct a chain of block. Correspondingly, peer nodes are liable for maintaining their ledger data. Besides, they verify transactions in the network and endorsing them. They also play an important role in communication between different consortia. In fact, the protocol in Fabric is specifically designed called gossip. Both communication between different consortia and data consistency among nodes in the Fabric blockchain network relies on consensus mechanism. Typically, the consensus mechanism in Fabric is Kafka or Raft, which is belong to CFT consensus mechanism with high TPS and relatively high data security. In addition to consensus mechanism, codes, aka smart contracts are a fundamental part of blockchain. Fabric provides a set of SDKs for developing common gateway interface (CGI) codes deployed outside the blockchain network. Once website users access a specific URL, CGI codes can execute corresponding smart contracts inside the blockchain network and receive data submitted by the browser to perform some business logic. There are three programming languages can be selected for developing smart contracts in Fabric. However, we choose golang rather than Java or Node.js to develop smart contracts because smart contracts developed by golang has better performance than those of other two programming languages^23^. Smart contracts in the system are divided into three main parts: sensors contracts, permission contracts and traceability contracts. Firstly, sensors contracts store the sensor data received by the CGI interface into the Fabric blockchain network and read out the sensor history data from the Fabric blockchain network. Secondly, the combination of permission contracts and traceability contracts enable the control of the each CCPs’ traceability data. The architecture of smart contracts is shown in Fig. 3. Besides, types of smart contracts and their roles are listed in the Table 1. Smart contracts do satisfy a majority of requirements of the blockchain-based traceability, but there are still some basic features of website like login or register that they are not good at. Therefore, an auxiliary programming framework such as web framework is required. The web framework used for this system is Gin, which is a lightweight framework in golang. Gin can easily bind the JSON data sent by the browser and forward them to the blockchain network by high-performance CGI interface.

**Table 1:** Smart contract list.

Transaction module	Instruction	Entity type	Role access control
Incubation management	ALL	Incubation data	Incubation personnel, administrators
Breeding management	ALL	Breeding data	Breeders, administrators
Slaughter management	ALL	Slaughter data	Slaughterers, administrators
Process management	ALL	Process data	Processors, administrators
Logistics management	ALL	Logistics data	Logistics personnel, administrators
Get temperature data	Read	Temperature data	Analyst, administrators
Get humidity data	Read	Humidity data	Analyst, administrators
Get ammonia data	Read	Ammonia data	Analyst, administrators
Get noise data	Read	Noise data	Analyst, administrators
Get traceability data	Read	Traceability data	All
Incubation personnel	Intercept	Bytes from CGI	N/A
Breeders	Intercept	Bytes from CGI	N/A
Logistics personnel	Intercept	Bytes from CGI	N/A
Slaughterers	Intercept	Bytes from CGI	N/A
Processors	Intercept	Bytes from CGI	N/A
Administrators	Intercept	Bytes from CGI	N/A

### Dataflow based on HACCP

**Figure 4:**
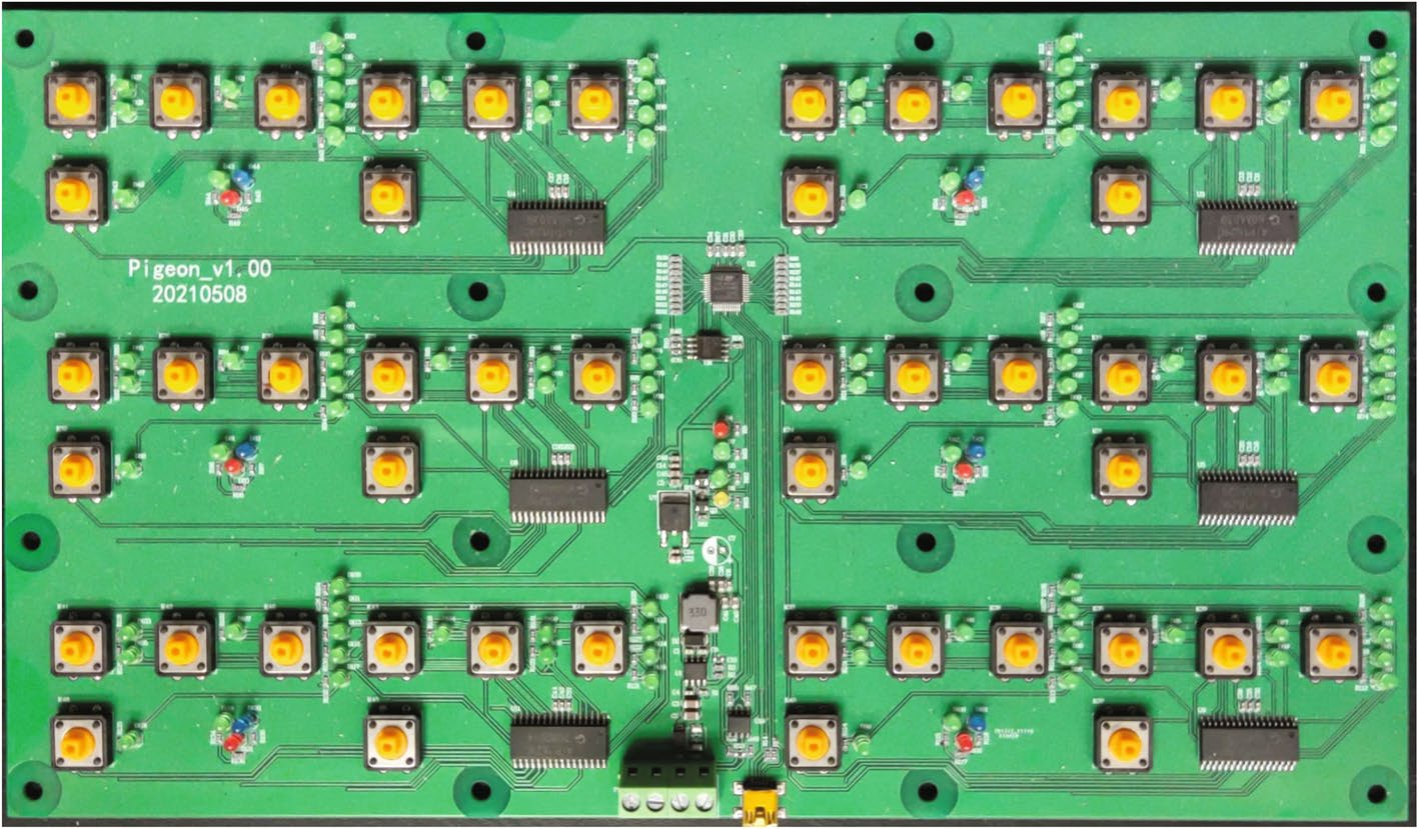
Circuit wafer.

**Figure 5:**
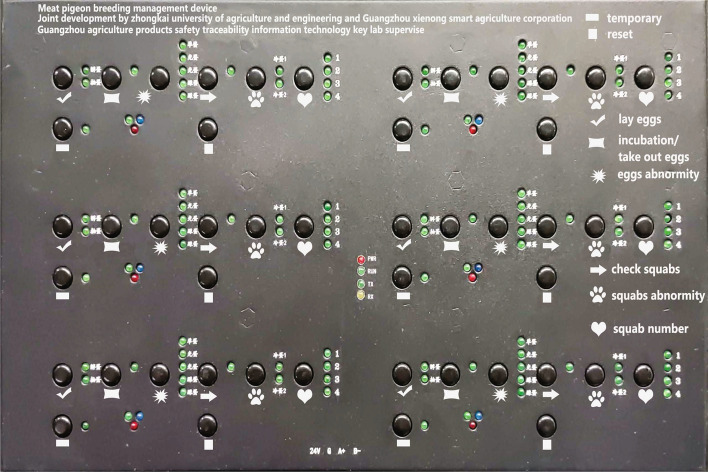
Farming information management devices.

Data in the system is mainly divided into two parts. The first part is various environment data, for example, temperature, humidity, ammonia concentration collected by sensors. With the help of DTU modules in sensors, data are transmitted via Zigbee protocol to the Fabric blockchain network. The second part is data gathered from CCPs in pigeon food production industry determined by HACCP principles. There are some web pages for implementing basic features of the system and data entry of each CCP. For instance, login page of the system is displayed in Fig. 2. Ultimately, data of every CCP are aggregated so that users can access the highly credible traceability data stored in the Fabric blockchain network through the traceability data query interface. The prime solution to simplify the complexity of traceability data which can be easily understood by user is HACCP principles. In our work, we make a lot of efforts to analyse pigeon food production industry. All critical control points (CCPs) determined by HACCP principles are described in detail in below stages:

- *Breeding*The breeding of pigeons is an important part of the pigeon food production industry with a link to the rest of the industry. The pigeon breeding environment is similar to a chicken farm. Thousands of pigeons are in a semi-enclosed loft of about 100 × 20 metres, with many cages put up side by side. The paired pigeons are placed evenly in the different cages and each pair takes care of 2–4 young pigeons. Different young pigeons may grow differently in size due to competitive feeding behaviour. Therefore, smaller young pigeons have to be switched manually with the larger ones in the other cages to avoid malnutrition or even death caused by hunger. Some pigeons’ duty is to look after squabs and others are responsible for laying eggs. Typically, each pair of pigeons lays two consecutive eggs at a regular intervals usually two days. After laying, breeders need to check the two eggs carefully. They will observe whether any egg is broken at first. Then they will shining a torch on eggs to ensure none of them is unfertilized. At last pigeon eggs pass inspection successfully will be sent to the hatchery for incubation. On the other hand, abnormal states of other pigeon eggs like broken or unfertilized will be recorded specifically. However, the majority of breeders are not familiar with computer because they usually have a low level of education, which leads to incorrect data entry sometimes. What’s worse, different workers who are used to using various names to record the abnormalities of the two pigeon eggs, which makes data entry in a mess. Besides, recording pigeon eggs’ state is a time-consuming and labor intensive job. In conclusion, it is required to conduct a reduction in force and make some improvements of recording pigeon eggs’ state data more accurate. Therefore, we design and manufacture a microcontroller with plastic shell as an auxiliary pigeon breeding information management device to generate uniform data of pigeon eggs’ abnormal state automatically instead of manual data entry. The device with shell is shown in Fig. 5 and the microcontroller inside it is shown in Fig. 4. Once breeders press the button corresponding to the pigeon egg states, generated data will be transferred to computer via a USB cable. Breeders are willing to use the device because it can make them get rid of recording data. To guarantee credibility of data generated by device, data will be sent to the Fabric blockchain network for storage by executing some well-written smart contracts specifically. Furthermore, data of pigeon eggs’ state are no longer chaotic due to automatically generated data in a standardised format. In our work, we use one byte to represent all possible states of two pigeon eggs. Table 2 describes the use of the high four bits of a byte to represent the different stages of a pigeon’s state in the cage and Table 3 lists the use of the low four bits of the byte to express the different abnormal states of pigeon eggs.

**Table 2:** Relationship between the pigeon cage and first 4 bits from State Byte.

Stage	First 4 bits from state byte
The pigeons in this cage are in the egg-laying stage	0 × 10
Checking the pigeon eggs in the cage for no fertilization or breakage with light (the light check needs to be done 3-5 days after the eggs are laid)	0 × 20
The eggs laid in this cage can be transferred to the hatchery	0 × 30

**Table 3:** Relationship between the eggs status and last 4 bits from State Byte.

Status	Last 4 bits from state byte
2 fertilized eggs	0 × ?0
Only one egg was laid	0 × ?1
1 unfertilized egg and 1 fertilized egg	0 × ?2
2 unfertilized eggs	0 × ?3
1 broken egg and 1 fertilized egg	0 × ?4
1 broken egg and 1 unfertilized egg	0 × ?5
2 broken eggs	0 × ?6

**Figure 6:**
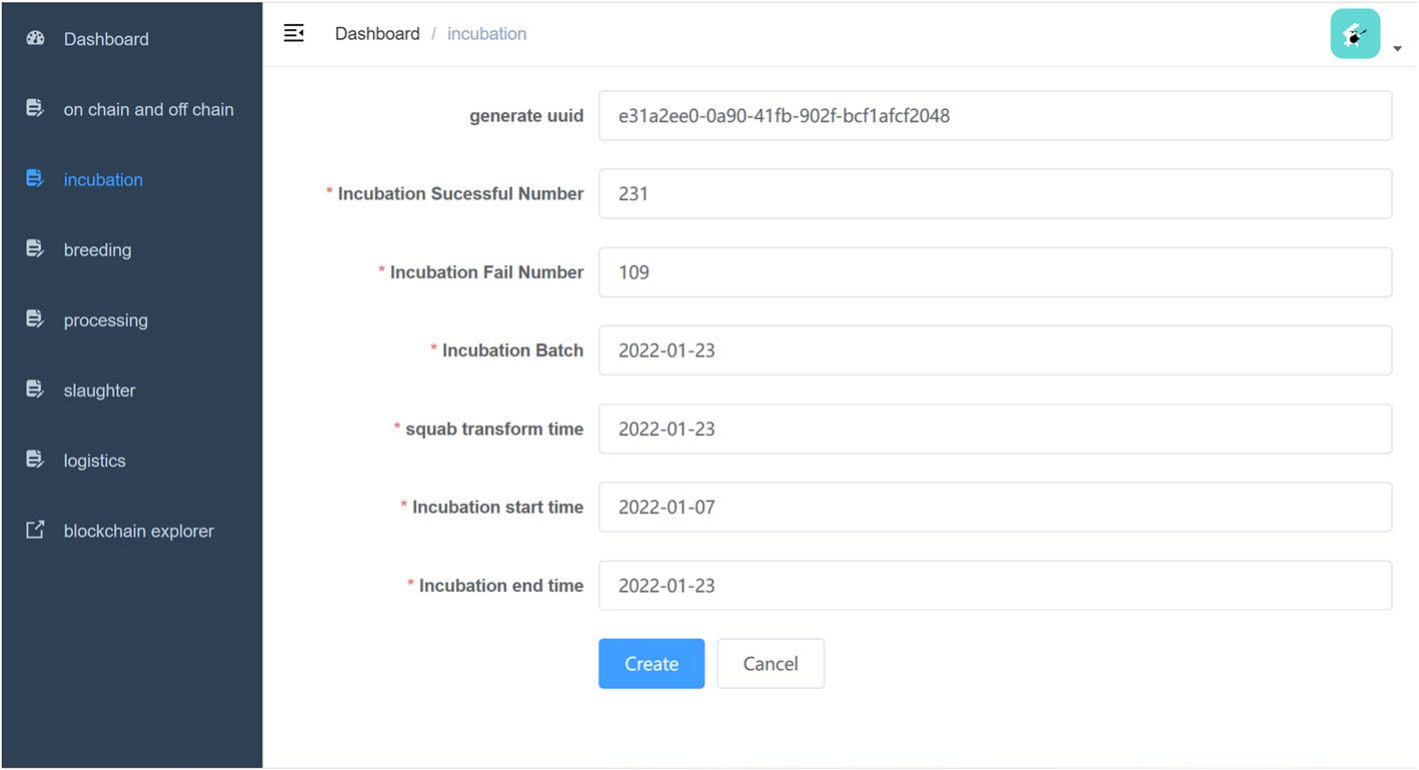
Incubation form.

- *Incubation* Incubation is the origin of pigeon food production industry. Similarly, hatching data can be treated as genesis data of pigeon food production industry. Generally, pigeon eggs incubation contains two phases. Firstly, pigeon eggs from the breeding factory will be clustered together and be centrally incubated in the hatchery. Secondly, squabs will be transferred to pigeon lofts. Incubation time and squab transfer time are important for traceability, so they will be recorded by smart contracts. Figure 6 demonstrates the incubation webpage of the website. Algorithm 1 describes the process of how new incubation data be recorded in the Fabric blockchain network.

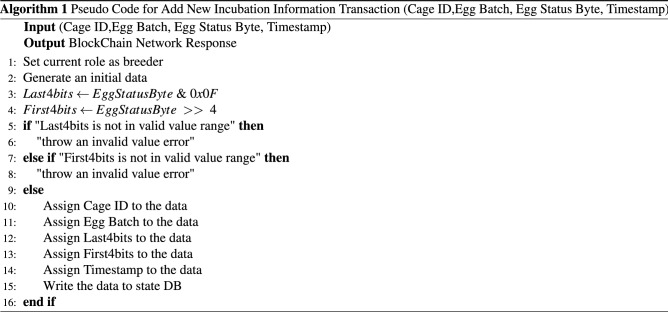

- *Slaughter* Slaughter refers to the process of producing the raw material of meat. Generally, pigeons have a finite number of eggs they would lay during their breeding lifetime. Consequently, when pigeons stop laying eggs, they will be marked with the corresponding batch ID according to the current date initially. Then they will be sent to the slaughterhouse for slaughter. Slaughter in modern slaughterhouse is orderly and fast. The residual meat of the slaughtered pigeons varies according to the individual and is generally classified as 250 g, 300 g or 350 g. In conclusion, weight of meat and slaughter batch ID are important for traceability in this CCP. Both of them will be recorded into blockchain by smart contracts. After slaughter, different weights of meat will be processed by different processing methods.
- *Processing* Processing of pigeon food involves many complex phases and every phase may need to add several food additives. Types and quantities of food additive added in different food production will be allocated according to actual market demand and the weight of the pigeon meat after slaughter. Any illegal food additives or unclean environment for processing food will result in food safety issues. In conclusion, as one of CCPs, processing might be the most important CCP for ensuring food safety. Therefore, we record every phases of pigeon food production process and food additives in each phase in detail by executing smart contracts specifically for traceability. This part of data can make users know what happened during food processing and what food additives have been added to food although they are not physically present.
- *Logistics* Logistics is the last CCP of the pigeon food production industry. logistics is also complex which involves many different locations and people. It is not an easy job for drivers to keep goods being in good condition during delivery. Negative factors like rough handling, crowded carriages and bad weather may cause food spoil or damage before it is delivered. Therefore, it is significant to record some logistics data like who is driver and where goods pass through by smart contracts. By logistics traceability data stored in blockchain with high credibility, people can easily make sure responsibility attribution when food safety issues happened.

## System implementation and performance analysis

### Introduction of system deployment environment

The Fabric blockchain network are composed of various Docker containers which deployed on an Alibaba Elastic Cloud Server (ECS). The server configuration environment is shown in Table 4. The Fabric blockchain network requires three necessary components to run successfully: Docker images, binary executable files, and a series of configuration files. Configuration files includes system core parameters configuration files and the Docker-Compose configuration files. The system core parameters configuration files define some basic system configuration parameters such as block size of blockchain, endorsement policies while the Docker-Compose configuration files define the topology of the Fabric blockchain network. This system uses Fabric 1.4.4 version Docker images, Fabric 1.4.4 version binary executable files and system core parameters configuration files. Each binary executable file has its individual functionality, for example, generating the node’s MSP identity material or generating the genesis block, aka the first block. Typically, there is a Linux shell script for initiating the Fabric blockchain network with configuration files and executable files. To boot the Fabric blockchain network successfully, paths where binary executable files and configuration files stored in computer needs to be added to the system’s environment variables so that the Linux shell script can be correctly executed by calling these binary executable files and reading other configuration files. To further increase security of the system, communication in both the Fabric blockchain network and the clients outside the network are transport layer security (TLS) encrypted.

**Table 4:** Server configuration.

Hardware	Configuration
CPU	Intel(R) Xeon(R) Platinum 8269CY CPU T 3.10 GHz
OS	CentOS
Memory	16 GB

The topology of Fabric blockchain network is fundamental for blockchain-based application. There is one order consortium, two peer consortia in the Fabric blockchain network in this system. From the perspective of nodes, three order nodes compose the order consortium and two peer nodes are in each peer consortium. Besides, there are two CA nodes in the network. The boot deployment process of Fabric blockchain network can be roughly divided into three stages. Firstly, the binary executable files are invoked to generate the membership service provider (MSP) identity certificate of every node. Then CA nodes verify the reliability of each node’s MSP identity certificate. Secondly, Docker downloads various Docker images rely on pre-written yaml format configuration files and generate various Docker containers based on corresponding Docker images to constitute the blockchain network. Thirdly, after booting the network, the Linux shell script generates a genesis block and a channel. Then order nodes and peer nodes of different consortia join to the channel in turns. After that the developed smart contracts are deployed in the blockchain network. More specifically, successful deployment of smart contracts in Fabric version 1.4.x need to go through two stages: install and instantiate. After a successful instantiation, the Fabric’s cli Docker container generates a Docker image based on the developed smart contracts. Then Docker uses the generated Docker image to create a Docker container to integrate smart contracts into the Fabric blockchain network.

### System interface

A RESTful API^24^ is an architectural style for an application program interface (API) which access and use data by HTTP requests. That data can be used to GET, PUT, POST and DELETE data types, which refers to the reading, updating, creating and deleting of operations concerning resources. Based on that style, we carefully design APIs of the system according to both HACCP principles and business requirements of pigeon food production industry, as shown in Table 5 below. For example, if we need to access noise data, we can employ the api name called 'getnoise' in Table 5. The detailed description can be designed by the software engineer according to actual business requirements.

**Table 5:** Restful API list.

Name	Action	Media type	Detail
incubationmanagement	ALL	Application/json	Manage incubation data
breedmanagement	ALL	Application/json	Manage breeding data
slaughtermanagement	ALL	Application/json	Manage slaughter data
processmanagement	ALL	Application/json	Manage process data
logisticsmanagement	ALL	Application/json	Manage logistics data
gettemperature	GET	Application/json	Access temperature data
gethumidity	GET	Application/json	Access humidity data
getammonia	GET	Application/json	Access ammonia data
getnoise	GET	Application/json	Access noise data
gettraceabilityinfo	GET	Application/json	Access traceability info

**Figure 7:**
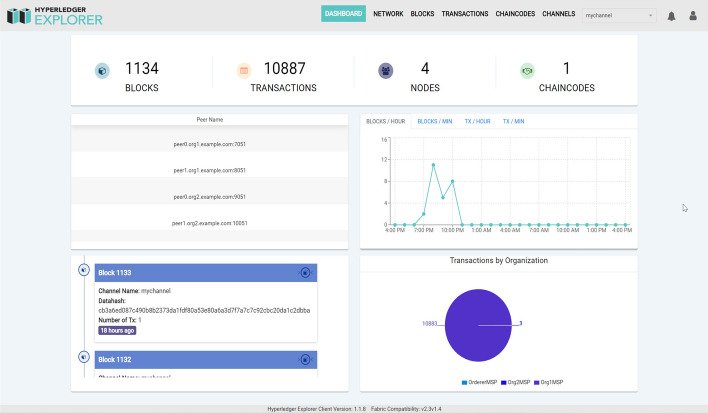
Blockchain explorer.

The regular work of RESTful APIs in our system depends on healthy running of the Fabric blockchain network. There are some key indicators, for instance, number of blocks, number of transactions, number of nodes, and number of consortium need to be monitored for keeping the Fabric blcokchain network healthy. To fulfill this need, the open-source community makes some efforts to develop some tools. One of the relatively famous ones is Hyperledger Explorer^25^ and a similar tool is also available in Ledgerdata Refiner^26^. To enable that users of the system can check status of the Fabric blockchain network at any time easily, Hyperledger Explorer is integrated into the system. The monitor webpage is shown in Fig. 7 below.

### Performance analysis

The system’s stability is critical to the user’s actual use experience. Typically, one solution to keep the system running stably as long as possible is doing some simulation like performance testing before the system is put into use. Performance testing^27^ tests the software system’s performance under various workloads by specific software. The aim of performance testing is to make sure that the system is reliable and should not crash or there should not be any blocking issues. There are two main metrics commonly used in performance testing: throughput and latency. Throughput refers to the number of HTTP requests sent by the system in a period of time. It can be divided into two main categories: read throughput and transaction throughput. Throughput limitations are an important manifestation of a performance bottleneck in software system. System throughput limitations data can assist users in locating system performance bottlenecks as soon as possible. Correspondingly, latency refers to the delay time between a HTTP request and a HTTP response which mainly includes two categories: read latency and transaction latency. It is significant to do some latency tests because too long delay will cause terrible use experience. In conclusion, the formulas for the four performance indicators mentioned above are listed below:

- Read Throughput = $$\frac{\text {The amount of total read operations}}{\text {Duration in total}}$$
- Transaction Throughput = $$\frac{\text {The amount of total valid transactions}}{\text {Duration in total} }$$
- Read Latency = Received time of response operation - Submission time of read operations
- Transaction Latency = Confirmation time network threshold - Submission time of transaction

**Figure 8:**
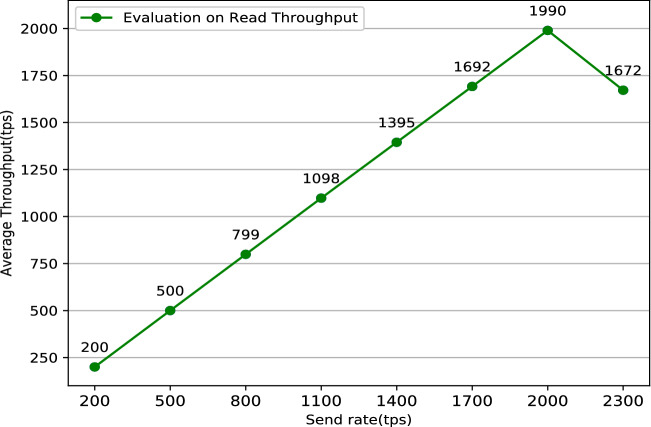
Evaluation on read throughput.

**Figure 9:**
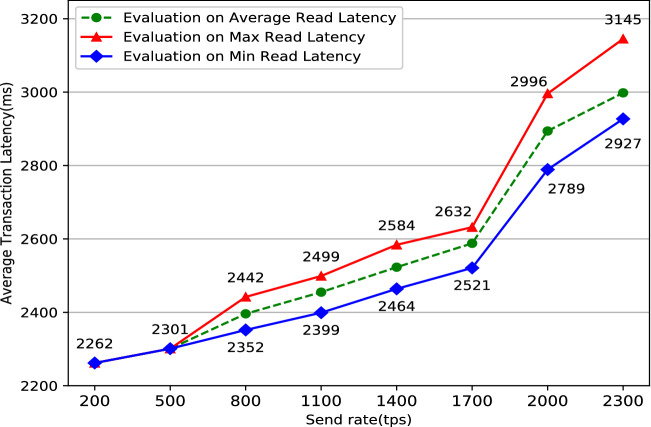
Evaluation on read latency.

**Figure 10:**
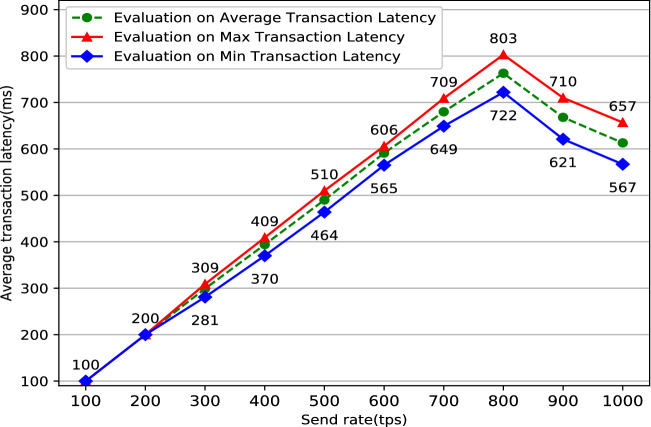
Evaluation on transaction latency.

**Figure 11:**
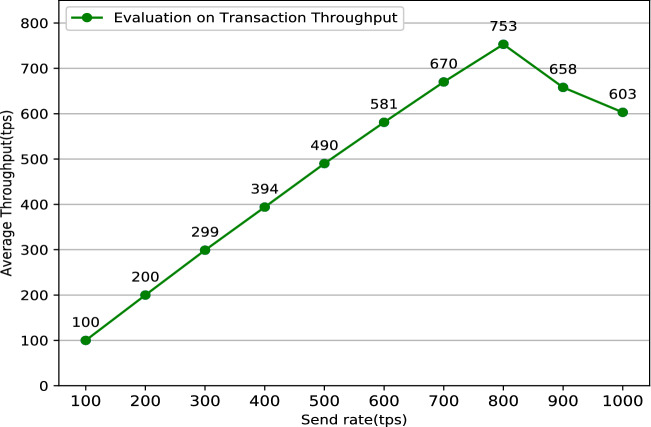
Evaluation on transaction throughput.

Performance testing in blockchain-based system especially in Fabric must be done with the help of specific performance testing tools. Caliper^28^ is a common performance testing tool designed specifically for the Fabric network, but it is cumbersome to configure and run. To simplify the performance testing processes for the Fabric network, Technical Working Group China (TWGC) has developed a lightweight performance testing tool called Tape^29^, which is much simpler and easier to use than Caliper. Jmeter^30^ is a versatile tool for websites’ performance testing. It can test different websites by modifying configuration files to achieve specific testing functions in the meanwhile. Some researches have been done by doing performance testing on different Fabric systems^31–33^, but it seems that there are some differences among different blockchain-based system with individual architecture. This research uses a combination of results from Tape and Jmeter as the final result. The performance test result such as read throughput, read latency, transaction latency, transaction throughput are shown in Figs. 8, 9, 10, 11.

## Results and discussion

### Contribution

The numerous processes in the pigeon food production industry make it difficult to regulate and prone to food safety crisis. To guarantee food safety, one effective method is to establish a food traceability system which is a typical application of agricultural information. However, traditional traceability system usually uses centralized databases to store traceability data with low credibility because centralized storage can be easily tampered with. Furthermore, data entry of traceability system requires a considerable number of manpower. Therefore, our work is focus on building a blockchain-based traceability system integrated with various hardware devices to improve deficiencies mentioned above. More precisely, we choose HACCP principles to analyse pigeon food production industry and determine some CCPs firstly, which decreases the complexity of the industry. Secondly,we develop some webpages based on these CCPs to gather traceability data of each CCP. Besides, we install sensors in pigeon loft for collecting environment data to monitor breeding environment. A stable breeding environment can reduce the risk of pigeon breeding. Furthermore, we produce an auxiliary device to generate pigeon eggs’ state data automatically instead of data entry manually. The device not only alleviates the coming workforce shortage because of ageing population but also reduce the workload of every breeder. Thirdly, we boot the Fabric blockchain network and store all traceability data into it. Therefore, all traceability data are highly credible and are hardly to be tampered with. However, there is few work about building a permissioned blockchain-based traceability system integrated with IoT for informatization in pigeon food production industry. Our work makes some contributions in this area.

### Limitations and future research directions

Initially, the number of consortia in the Fabric blockchain network and the number of nodes in each consortium are configured by the configuration file of Docker-Compose. If there are some requirements to change the topology of the network, the administrator of the blockchain-based system must modify the configuration file and restart the network. However, system is not available during this period. Another deficiency is that the deployment and migration of the Fabric blockchain network is still complex because they relies heavily on running the logic in a Linux shell script. Moreover, if a system migration is required, there are also a number of file paths in the configuration file that need to be changed. Therefore, the future solution is implementing the blockchain as a service (BaaS) functionality to improve these deficiencies. Another gap is the environmental data collected by the sensors still lacks a model for data analysis. The next plan is to study machine learning algorithms and choose suitable algorithms such as the RNN^34^ family of LSTM^35–37^ to process the environmental data and predict possible future environmental anomalies. Currently the system uses an auxiliary information management device to generate data by pressing buttons instead of manual data entry. However, manual data entry is still used in other CCPs of the pigeon food production industry, which is labor intensive and may lead to data entry errors. It is planned to use some other hardware to minimize errors in manual data entry and reduce the workload of data entry for every worker. Besides, there are some new types of blockchain, for example, directed acyclic graph (DAG) typically represented by IOTA^38^. It has its specific advantages in terms of TPS^39^, which should be paid more attention in the future. Moreover, security of blockchain application is worth attaching great importance as well^40^.

## Conclusion

Pigeons go through many complex stages from hatching to processed meat products for delivery to consumers. But negligence in any one of stages will lead to food safety issues and result in a crisis of confidence among consumers. Therefore, it is an important and significant research topic to apply a variety of hardware and software technologies with the purpose of improving both productivity of agricultural production and safety of poultry food production. In addition, comprehensive use of software and hardware technologies can also reduce the necessary amount of workers and workload of each worker in poultry food production industry to decrease the adverse impact of population ageing. Based on the HACCP principles, this research carefully investigates the pigeon food production industry and determines the five critical control points (hatching, breeding, slaughtering, processing, and logistics) as a guiding ideology to construct a traceability system. From the perspective of architecture, the traceability system integrated various techniques. Firstly, the storage module of this system is one of permissioned blockchain, Fabric, with the advantages of guaranteeing data security and keeping relatively high TPS even suffering huge number of website accessing. Secondly, the system contains a variety of sensors which can collect various environment data and monitor the environment relying on these data. Thirdly, to further reduce workload of workers especially breeders, we design a microcontroller device with buttons which can generate data by pressing different buttons standing for corresponding state of a pair of pigeon eggs laid by one female pigeon. To summarize, the system not only improves agricultural production efficiency but also alleviates the shortage of agricultural workforce caused by an ageing population. Furthermore, it promotes the development of the pigeon food production industry. In conclusion, the system is an exploration of a new generation of distributed and trusted storage technology combined with hardware in the field of pigeon food production industry.

## Data Availability

The datasets used and/or analysed during the current study available from the corresponding author on reasonable request.
